# TIGAR2: sensitive and accurate estimation of transcript isoform expression with longer RNA-Seq reads

**DOI:** 10.1186/1471-2164-15-S10-S5

**Published:** 2014-12-12

**Authors:** Naoki Nariai, Kaname Kojima, Takahiro Mimori, Yukuto Sato, Yosuke Kawai, Yumi Yamaguchi-Kabata, Masao Nagasaki

**Affiliations:** 1 Department of Integrative Genomics, Tohoku Medical Megabank Organization, Tohoku University, 2-1 Seiryo-machi, Aoba-ku, Sendai, Miyagi, 980-8573, Japan

**Keywords:** Transcript isoform quantification, RNA-Seq, variational Bayesian inference, graphical models

## Abstract

**Background:**

High-throughput RNA sequencing (RNA-Seq) enables quantification and identification of transcripts at single-base resolution. Recently, longer sequence reads become available thanks to the development of new types of sequencing technologies as well as improvements in chemical reagents for the Next Generation Sequencers. Although several computational methods have been proposed for quantifying gene expression levels from RNA-Seq data, they are not sufficiently optimized for longer reads (e.g. > 250 bp).

**Results:**

We propose TIGAR2, a statistical method for quantifying transcript isoforms from fixed and variable length RNA-Seq data. Our method models substitution, deletion, and insertion errors of sequencers based on gapped-alignments of reads to the reference cDNA sequences so that sensitive read-aligners such as Bowtie2 and BWA-MEM are effectively incorporated in our pipeline. Also, a heuristic algorithm is implemented in variational Bayesian inference for faster computation. We apply TIGAR2 to both simulation data and real data of human samples and evaluate performance of transcript quantification with TIGAR2 in comparison to existing methods.

**Conclusions:**

TIGAR2 is a sensitive and accurate tool for quantifying transcript isoform abundances from RNA-Seq data. Our method performs better than existing methods for the fixed-length reads (100 bp, 250 bp, 500 bp, and 1000 bp of both single-end and paired-end) and variable-length reads, especially for reads longer than 250 bp.

## Background

Massively parallel sequencing of cDNA libraries constructed from RNA samples (RNA-Seq) has become a popular choice for quantifying gene expression levels of transcript isoforms [1]. Advantages of RNA-Seq over conventional microarray technologies include its larger dynamic range for quantification and capacity of identifying novel isoforms at one nucleotide resolution without the need for designing cDNA probes. A typical RNA-Seq data analysis workflow consists of two components: aligning sequenced reads to the reference cDNA sequences, and quantifying transcript isoform abundances based on the number of mapped reads on the reference sequences. In measuring gene expression levels, FPKM (Fragments Per Kilobase of transcript per Million mapped reads) is calculated under the assumption that a relative expression level of an isoform is proportional to the number of cDNA fragments that originate from it [2].

Since reads are typically 50-300 bp paired-end for Illumina sequencers, in many cases, they can be aligned to more than one isoform and/or locations on the reference sequences. One of challenges for accurate estimation of gene expression is to handle such multi-mapped reads [3]. Several approaches have been proposed to model uncertainty of read mappings in a probabilistic framework, and it has been shown that the statistical inference of read mapping is effective for more accurate estimation of gene expression levels [4,5]. Although rigorous simulation analyses with various conditions (such as 35 bp vs. 70 bp, and single-end vs. paired-end data) have been performed with several tools in the literature [6], cases for longer reads, such as 250 bp or longer that can be produced from the latest Illumina MiSeq sequencer, have not been extensively studied so far. Moreover, there are few methods suitable for RNA-Seq data produced from new types of sequencers, such as the Ion Torrent PGM sequencer, which generate variable-length reads with relatively higher error rate of substitutions, deletions, and insertions [7,8].

In this paper, we present a statistical method, TIGAR2, which implements new features for improving sensitivity and accuracy of quantification of isoform expression levels from RNA-Seq data by extending the originally developed method [5]. First, for achieving maximum sensitivity for mapping longer reads to reference sequences, TIGAR2 can handle aligned reads from BWA-MEM [9], as well as other widely used alignment tools such as Bowtie2 [10]. Sequencing errors (substitutions, deletions and insertions) within reads that can be inferred from the gapped alignments of reads to reference sequences are modelled under a probabilistic framework in TIGAR2. Second, in order to speed up the variational Bayesian inference in TIGAR2, a new algorithm is implemented so that only reads that can influence isoform abundance parameters in the next iteration are detected and considered in the following update equations.

In order to evaluate quantification performance with TIGAR2, we prepare simulation data that emulates Illumina fixed-length reads (both single-end and paired-end) and Ion Torrent variable-length reads data. For simulating the variable-length reads, a variable read length distribution is empirically estimated from the actual RNA-Seq data by non-parametric regression with Gaussian kernels as basis functions in our analysis. We also apply TIGAR2 to real data of human cell line samples and evaluate consistency of estimated gene expression levels among technical replicates.

## Methods

**Figure 1:**
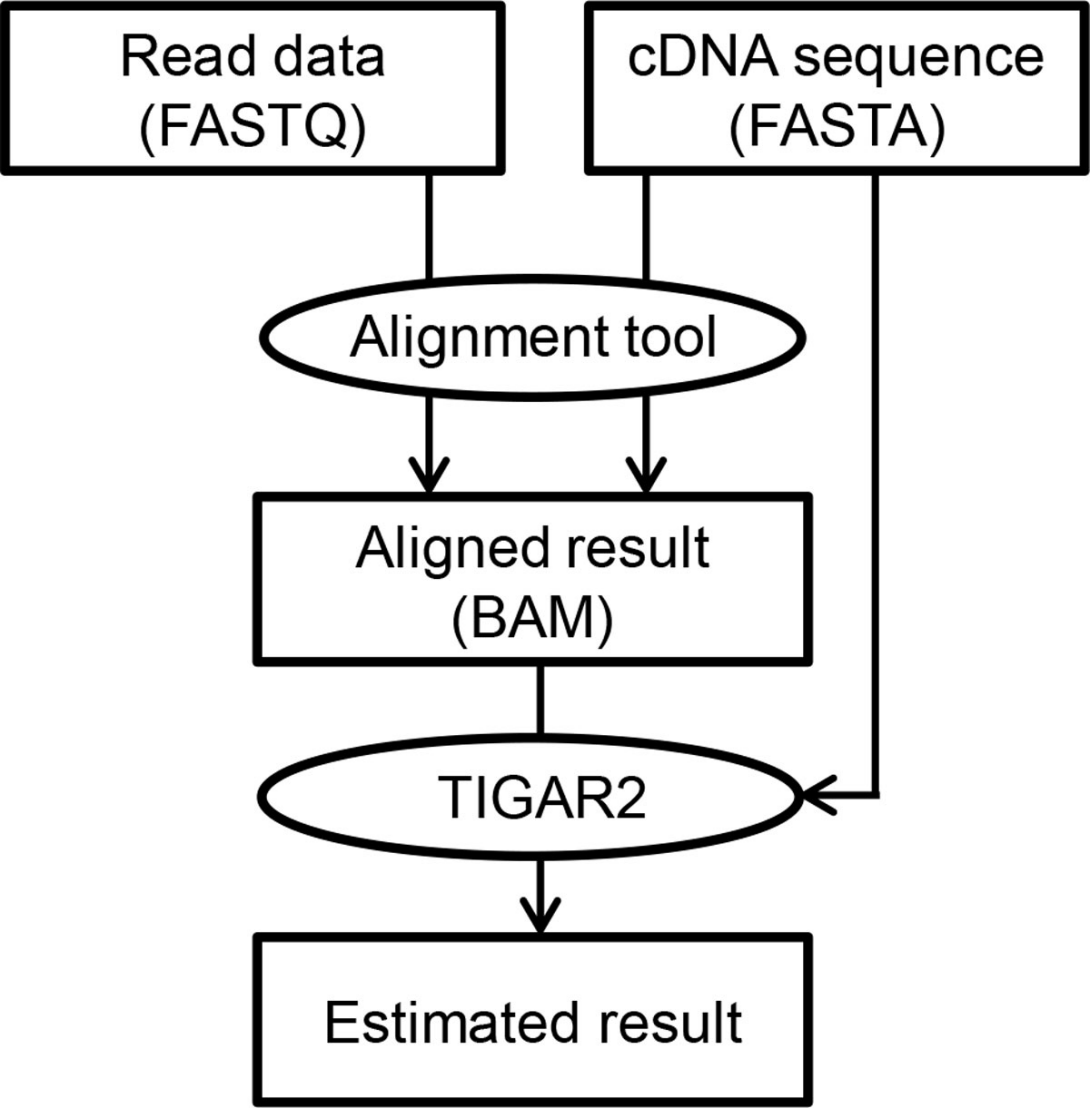
**The TIGAR2 pipeline**. The schematic diagram shows a typical workflow of running TIGAR2 software. Alignment tools such as Bowtie2 take two input files, read data in FASTQ format and cDNA reference sequences in FASTA format. After reads are aligned to the cDNA sequence, the generated BAM file and the reference FASTA file are used in TIGAR2 for estimating transcript isoform abundances and calculating FPKMs.

A pipeline of running TIGAR2 consists of two steps: alignment of reads to reference sequences, and estimation of transcript isoform abundances based on the alignment result (Figure 1). Since the first part of the pipeline uses external alignment tools for aligning reads to the reference sequences, it is recommended to run the whole pipeline in the UNIX environment. Details of each step are described in the following sections.

### Alignment of reads to reference sequences

Reference cDNA sequences in the FASTA format of model organisms are either available from the RefSeq database [11], or can be generated from the whole genome reference sequence and a gene annotation file (GTF format) with a tool called "gffread", which is included in the Cufflinks package [2]. For cases of non-model organisms, de novo transcriptome assembly might be considered [12], and then the resulting contigs can be used as reference sequences. Given a set of cDNA sequences in FASTA format, the FM-index for the following alignment step is constructed with the corresponding alignment tool. Then, gapped-alignments of reads to the reference sequences are generated with Bowtie2 or BWA-MEM with allowing multiple mappings of reads to the reference cDNA sequences.

### Generative model of RNA-Seq data

**Figure 2:**
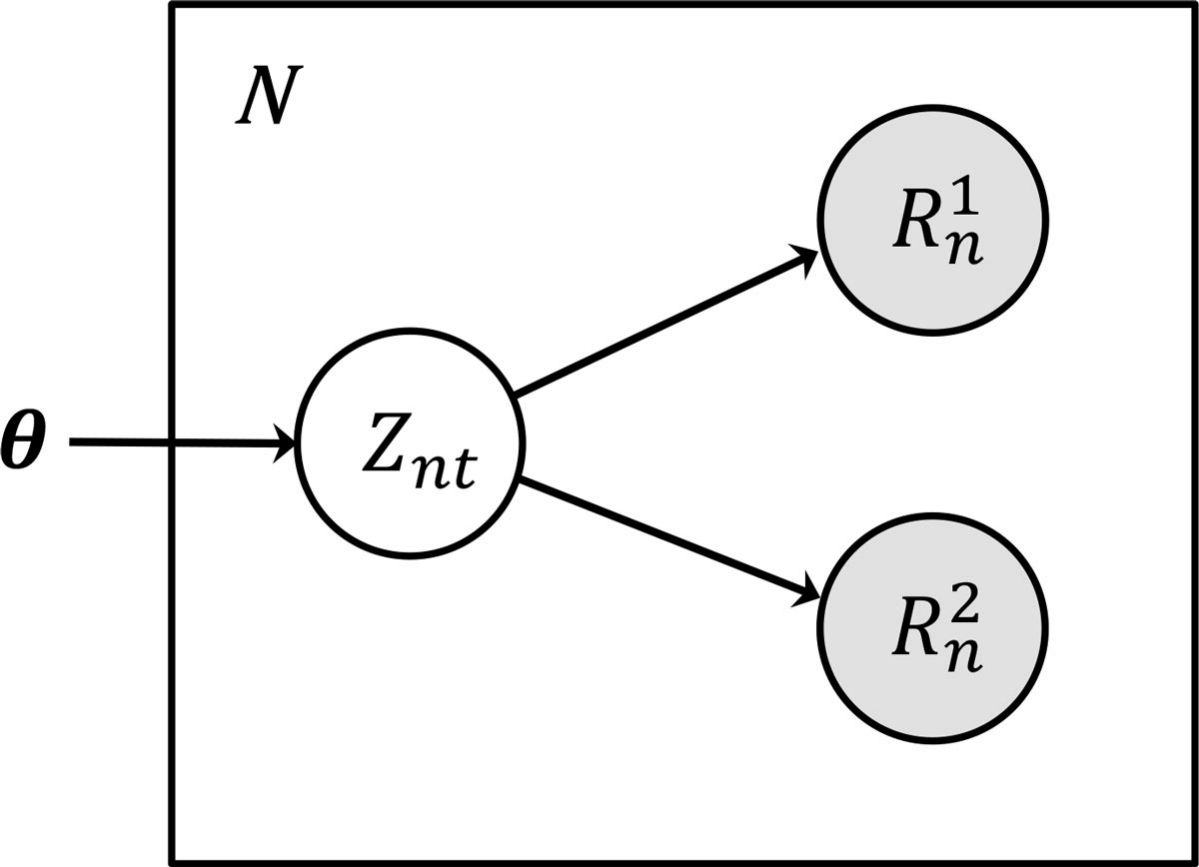
**The generative model for RNA-Seq data in TIGAR2**. The transcript isoform abundance parameter, indicator variable for transcript isoform choice, nucleotide sequence of the first and second pair read  n are represented by  θ, $Z_{nt},R_{n}^{1},$ and $R_{n}^{2}$, respectively.

After the alignment is complete, TIGAR2 takes the resulting SAM/BAM and the FASTA files as input for transcript isoform abundance estimation. We use a generative model for RNA-Seq data as described in Figure 2, which is an extended version of the original model [5]. Here,  θ is a model parameter that represents transcript isoform abundances, and $Z_{nt}$ is an indicator variable and it takes one if read  n is generated from transcript isoform  t, and zero otherwise. $R_{n}^{1}$ and $R_{n}^{2}$ are the nucleotide sequence of the first and second pair of read *n*, respectively. Then, the joint probability of the model is decomposed as the product of conditional probabilities as follows:

$$P\left(\theta ,Z_{nt},R_{n}^{1},R_{n}^{2}\right)=P\left(\theta \right)P\left(Z_{nt}\text{|}\theta \right)P\left(R_{n}^{1},R_{n}^{2}\text{|}Z_{nt}\right).$$

*P *( θ) is the prior distribution of the parameter and we assume the Dirichlet distribution:

$$P\left(\theta \right)=\frac{1}{C}\overset{T}{\underset{t=0}{\prod }}\theta_{t}^{\alpha_{t}-1},$$

where $\alpha_{t}>0$ is a hyperparameter,  C is a constant,  T is the number of transcript isoforms, and ${\text{Σ}}_{t=0}^{T}\;\theta_{t}=1$. Here, $\theta_{0}$ represents the noise isoform abundance (reads that are not generated from any known isoform are assigned).

$P\left(Z_{nt}\text{|}\theta \right)$ is the conditional probability of $Z_{nt}$ given  θ and we further decompose as follows:

$$P\left(Z_{nt}\text{|}\theta \right)=P\left(T_{n}\text{|}\theta \right)P\left(F_{n}\text{|}T_{n}\right)P\left(S_{n}\text{|}T_{n},F_{n}\right)P\left(O_{n}\text{|}T_{n}\right)P\left(A_{n}^{1},A_{n}^{2}\text{|}T_{n},F_{n},S_{n},O_{n}\right),$$

where $T_{n},F_{n},S_{n},O_{n}$, $A_{n}^{1}$, and $A_{n}^{2}$ respectively represent the transcript isoform choice, fragment size, read start position, orientation, and alignment state of the first pair and second pair of read  n. $P(T_{n}|\theta )$ represents the probability of read *n *generated from transcript isoform $T_{n}$ given a parameter vector, and we compute $P(T_{n}=t|\theta )=\theta_{t}$. Compared to the original model of TIGAR [5], a fragment size variable is now included in the model. The conditional probability of observing $F_{n}=f_{n}$ given $T_{n}=t_{n}$ is calculated by truncated and normalized distribution [6,13,14]:

$$P\left(F_{n}=f_{n}\text{|}T_{n}=t\right)=\frac{d_{F}\left(f_{n}\right)}{\sum_{x=1}^{l_{t}}d_{F}\left(x\right)},$$

Where $l_{t}$ is the length of transcript isoform  t, and $d_{F}\left(x\right)$ is the global fragment size distribution. We construct $d_{F}\left(x\right)$ based on the normal distribution with mean *μ_F _*and standard deviation *σ_F_*, which can be either specified according to experimental protocols, or can be estimated from the primary alignments of reads for the case of paired-end data. $P\left(S_{n}\text{|}T_{n},F_{n}\right)$ represents the probability of the start position of the first pair of read  n given the transcript isoform choice and fragment size, and calculate $P\left(S_{n}=s\text{|}T_{n}=t\right)=1/f_{t}$ if mRNAs have poly(A) tails, and $P\left(S_{n}=s\text{|}T_{n}=t\right)=1/\left(f_{t}-L+1\right)$ if mRNAs do not have poly(A) tails. $P\left(O_{n}\text{|}T_{n}\right)$ represents the probability of the orientation of read  n given the transcript isoform choice. For a strand specific protocol, it can be set as $P\left(O_{n}=0\text{|}T_{n}=t\right)=1$ and $P\left(O_{n}=0\text{|}T_{n}=t\right)=0$. Otherwise, it can be automatically estimated from the primary alignment of reads from the RNA-Seq data. $P\left(A_{n}^{1},A_{n}^{2}\text{|}T_{n},F_{n},S_{n},O_{n}\right)$represents the probability of the alignment state of read  n given the transcript isoform choice, fragment size, start position, and orientation of read  n. The transition probability of the alignment state is calculated as described previously [5].

Finally, $P\left(R_{n}^{1},R_{n}^{2}\text{|}Z_{nt}=1\right)$ is the conditional probability of sequence of the first and second pair of read  n given $Z_{nt}=1$. We calculate this probability considering the observed read length as

$$P\left(R_{n}^{1},R_{n}^{2}|Z_{nt}=1\right)$$

$$=\overset{X^{1}}{\underset{x=1}{\prod }}emit\left(r^{1}\left[x\right],q^{1}\left[x\right],c^{1}\left[x\right],a^{1}\left[x\right]\right)\overset{X^{2}}{\underset{x=1}{\prod }}emit\left(r^{2}\left[x\right],q^{2}\left[x\right],c^{2}\left[x\right],a^{2}\left[x\right]\right),$$

where *emit *$\left(r^{1}\left[x\right],q^{1}\left[x\right],c^{1}\left[x\right],a^{1}\left[x\right]\right)$ is the emission probability of nucleotide characters of the first pair of read  n, $r^{1}\left[x\right]$ is the nucleotide character, $q^{1}\left[x\right]$ is the base call quality score, $c^{1}\left[x\right]$ is the nucleotide character of the corresponding reference sequence, $a^{1}\left[x\right]$ is the alignment state of the first pair of read  n at position  x. *emit *$\left(r^{2}\left[x\right],q^{2}\left[x\right],c^{2}\left[x\right],a^{2}\left[x\right]\right)$ is similarly calculated as for the first pair of the read.

### Modelling of variable read length distribution

Some sequencers, such as Ion Torrent PGM, produce reads whose lengths are variable. In order to simulate such variable read length, we model the conditional probability of the read length given the fragment size, which is also calculated by the truncated distribution [4]

$$P\left(L_{n}=length\;\left(R_{n}\right)|F_{n}=f_{n}\right)=\frac{d_{R}\left(length\left(R_{n}\right)\right)}{\sum_{x=1}^{f_{n}}d_{R}\left(x\right)},$$

where *length *$\left(R_{n}\right)$ is the observed length of read  n, and $d_{R}\left(x\right)$ is the global read length distribution. Here, $d_{R}\left(x\right)$ can be constructed based on a linear combination of the smooth functions by fitting it to the data in a non-parametric manner with *M *equally spaced Gaussian kernels as basis functions. Let

$$g\left(x\right)=\overset{M}{\underset{i=1}{\sum }}a_{i}m_{i}\left(x\right),$$

where $a_{i}$ is the coefficient parameter, and $m_{i}(x)$ is the normal distribution with mean *μ_i _*and standard deviation *σ*. From the RNA-Seq data, observations of read lengths and their frequency, $\left(x_{n},y_{n}\right)$, are constructed, where $x_{n}$ is the read length, and is $y_{n}$ the frequency of $x_{n}$, and ${\text{Σ}}_{n=1}^{N}\;y_{n}=1$. Then, the least squares estimate (LSE) of the parameter vector $a={\left(a_{1},\ldots ,a_{M}\right)}^{T}$ is obtained by

$$â=\text{arg}\underset{a}{\text{min}}\overset{N}{\underset{n=1}{\sum }}{\left\{y_{n}-g\left(x_{n}\right)\right\}}^{2}.$$

Define a real value matrix ${\text{B}}_{ij}=m_{j}(x_{i})$. Then, the ordinary LSE is calculated by

$$â={\left(B^{T}B\right)}^{-1}B^{T}y.$$

Then, the global read length distribution $d_{R}\left(x\right)$ can be constructed from g(x) as:

$$d_{R}\left(x\right)=\frac{g\left(x\right)}{{\text{Σ}}_{x\prime =1}^{\text{max}\left(L\right)}g\left(x\prime \right)},$$

where max(L) is the maximum read length of the read data.

**Figure 3:**
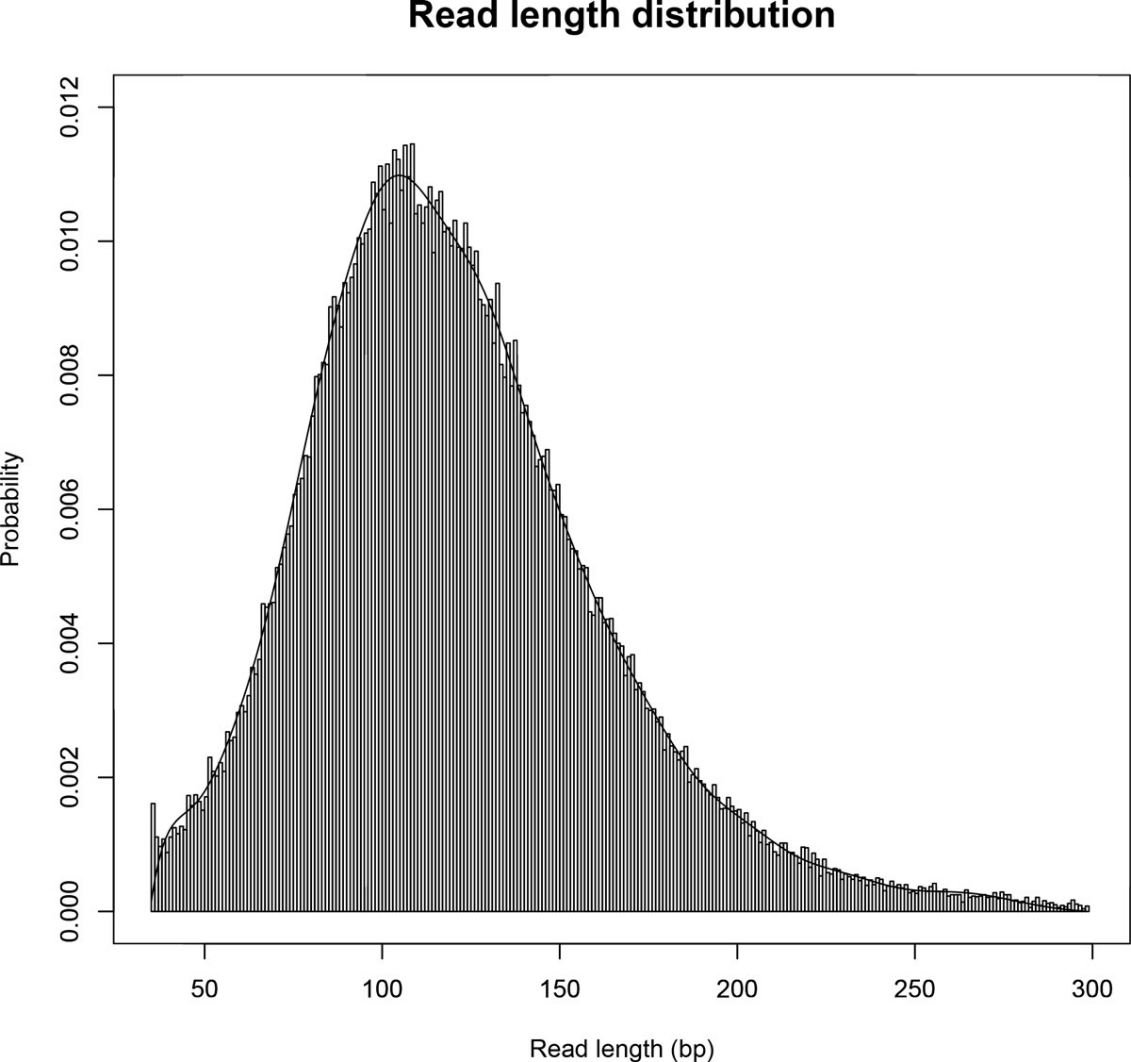
**Estimated read length distribution from the Ion Torrent PGM RNA-Seq data**. From the histogram of variable read lengths, the smoothly fitted probability distribution is constructed from a linear combination of 20 equally spaced Gaussian distributions(standard deviations are set to 20 bp) that minimizes the least squares error of the estimate.

An example of the estimated read length distribution from the real sequencing data of a human cell line (HeLa) sequenced by the Ion Torrent PGM sequencer (http://ioncommunity.lifetechnologies.com) is shown in Figure 3.

### Estimation of transcript isoform abundances

In our variational Bayesian inference approach, latent variables (true alignments of reads) as well as model parameters (transcript isoform abundances) are estimated as the posterior distribution. We use the Dirichlet distribution for the prior distribution $\theta \sim D\left(\alpha_{0},\ldots ,\alpha_{0}\right)$

with a single hyperparameter $\alpha_{0}>0.$ For $\alpha_{0}<1$, the prior favors solutions in which some of isoforms have zero abundance. Hence, $\alpha_{0}$ controls the complexity of model parameters (the number of possible transcript isoforms). A hyperparameter $\alpha_{0}$ is selected as a maximizer of the lower bound of the marginal log likelihood of the observed data. Here, we consider $\alpha_{0}$ = 0.001, 0.01, 0.1, or 1.0. Each iteration step of the variational approximation updates posterior distribution until a convergence criterion is satisfied. In the VBE step, the expected number of reads that are mapped to the transcript isoform *t *is obtained by $\hat{r_{t}}={\text{Σ}}_{n}E_{Z}\left[Z_{nt}=1\right]$. In the VBM step, the expected abundance of transcript isoform *t *is obtained by $E_{\theta }[{\hat{\theta }}_{t}]={\hat{\alpha }}_{t}/(\Sigma_{t},\hat{\alpha_{t\prime }})$, where $\hat{\alpha_{t}}=\alpha_{0}+\hat{r_{t}}$. Details of these update equations and calculation of the lower bound of the marginal likelihood are described in [5]. Recently, it has been shown that the variational inference described here is accurate in estimating the mean of posterior transcript expression, but not the variance [15].

The bottleneck of the computational cost of the inference algorithm is the calculation of $E_{z}\left[Z_{nt}=1\right]$ for all the possible alignments in the VBE step, which takes *O*(*M*) time if the total number of possible alignments is *M*. This time complexity is upper bounded by *O*(*NT*), where *N *is the number of reads and *T *is the number of cDNA reference sequences. Suppose some $E_{\theta }\left[{\hat{\theta }}_{t}\right]$ are already converged (unchanged from the previous iteration step) at the current step. We store the information in a Boolean variable *theta_converged *[*t*], which takes *true *if $E_{\theta }\left[{\hat{\theta }}_{t}\right]$ is converged, and *false *otherwise for each isoform *t*. Let $\tau_{n}$ be a set of isoforms to which read *n *is aligned. In the next VBE step, for each read *n*, $E_{z}[Z_{nt}=1]$ will not change if *theata_converged *[*t*] is *true *for all $t\in \tau_{n}$. To represent this information, we introduce a Boolean variable read_movable[n], which takes *false *if $E_{z}[Z_{nt}=1]$ will not change in the next VBE step, and *true *otherwise. The following algorithm computes read_movable[n] at the start of each iteration:

1. For each *t*, set *theta_converged *[*t*] to *true *if $E_{\theta }\left[{\hat{\theta }}_{t}\right]$ did not change from the previous step, and *false *otherwise.

2. For each *n*, if *theta_converged *[*t*] is *true *for all $t\in \tau_{n}$, then set read_movable[n] to *false*, and *true *otherwise.

Then, in the VBE step, *E_z_*[*Z_nt _*= 1] is computed where read_movable[n] is *true*. The algorithm heuristically eliminates unnecessary calculations of $E_{z}[Z_{nt}=1]$ drastically in the later part of iterations, in which most of $E_{\theta }\left[{\hat{\theta }}_{t}\right]$ are already converged and only a fraction of reads should be considered for calculating the update equations.

## Results and discussion

### Simulation data analysis

**Figure 4:**
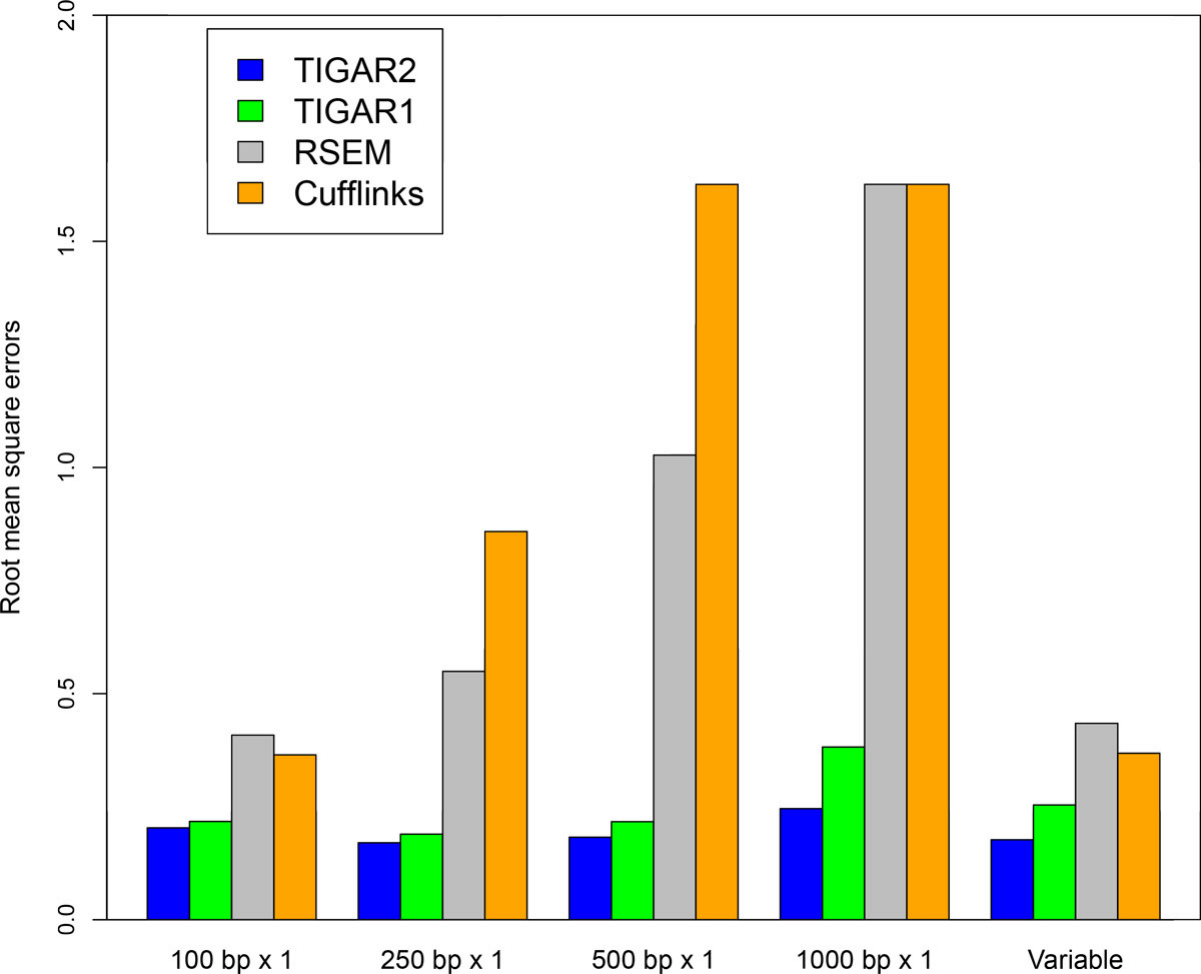
**Performance evaluation with TIGAR2, TIGAR1, RSEM, and Cufflinks using single-end and variable lengths simulation data**. Root mean square errors of the predicted transcript isoform abundances with each method against the true gene expression levels are shown for 100 bp, 250 bp, 500 bp, and 1,000 bp single-end, and variable-length simulation data. Because RSEM did not produce predictions for 1,000 bp single-end reads, errors were calculated assuming abundances were estimated as zero for all isoforms.

**Figure 5:**
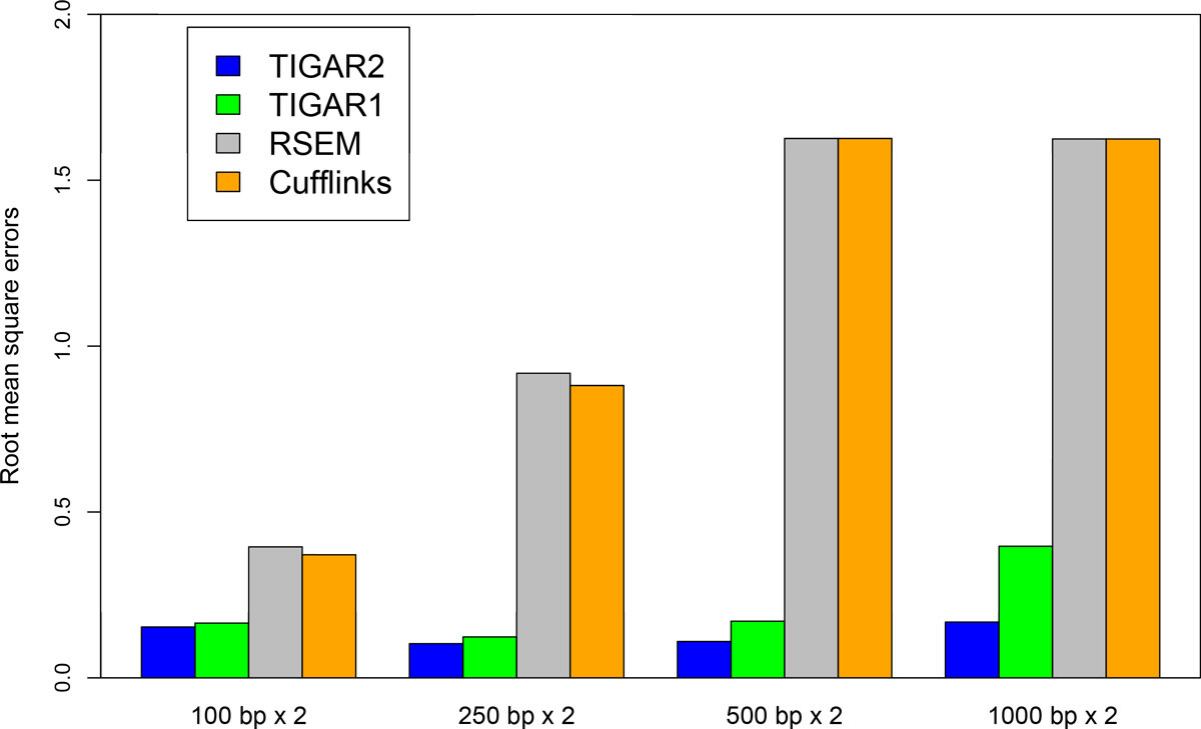
**Performance evaluation with TIGAR2, TIGAR1, RSEM, and Cufflinks using paired-end simulation data**. Root mean square errors of the predicted transcript isoform abundances with each method against the true gene expression levels are shown for 100 bp, 250 bp, 500 bp, and 1,000 bp paired-end simulation data. Because RSEM and TopHat-Cufflinks did not produce predictions for 500 bp and 1,000 bp paired-end reads, errors were calculated assuming abundances were estimated as zero for all isoforms.

We evaluate the performance of quantifying gene expression levels with TIGAR2 compared to existing methods using simulation data. First, 10,000 transcript isoforms in the human RefSeq database [11] are randomly chosen. Second, a set of true gene expression levels is constructed, in which log of isoform abundance is sampled from the standard normal distribution. Then, we generated 20 million, 8 million, 4 million, and 2 million RNA-Seq single-end reads of 100 bp, 250 bp, 500 bp, and 1000 bp, respectively, so that the total throughput of nucleotides remains the same. Similarly, 10 million, 4 million, 2 million, and 1 million paired-end reads of 100 bp, 250 bp, 500 bp, and 1000 bp, respectively, have been generated whose fragment size follows the normal distribution with *μ_F _*= 300, 750, 1250, and 2500, and *σ_F _*= 40, 100, 200, and 400, respectively. In order to simulate sequencing errors, we prepared a set of simulation data with 1% substitution, 1% deletion, and 1% insertion errors. All the simulation data was generated by our in-house software. After aligning reads to the reference cDNA sequences with Bowtie2 (the maximum number of allowed alignments per read is 100), transcript isoform abundances are estimated with TIGAR2. For comparing the performance, TIGAR1 [5], RSEM v1.2.10 [6] and Cufflinks v2.1.1 (with default options except '-u' and '-G' options) [2] are applied to the same simulation data. Although BitSeq [16] is also a relevant method, it is not included in our experiment since performance comparison with TIGAR was already conducted in their analysis [17]. Similarly, variable-length reads are generated according to the estimated read length distribution as shown in Figure 3, and isoform expression levels are estimated with each method. The root mean square errors of the estimated abundances (log of FPKMs) compared to the true gene expression levels are calculated and shown in Figure 4 and 5. For both fixed-length (single-end and paired-end) and variable-length reads, TIGAR2 consistently performed better than others. Especially, when read lengths > 250bp, the prediction accuracies with TIGAR2 over those with RSEM and Cufflinks are markedly better, which can be explained by more sensitive mapping with the latest alignment tools and efficient optimization of multi-mapped reads by the variational Bayesian inference implemented in TIGAR2. Since RSEM uses Bowtie as an aligner in the integrated pipeline, it becomes more difficult to align longer reads to the reference sequences without gapped-alignments of the reads, which potentially loses sensitivity of mappings.

### Real data analysis

**Figure 6:**
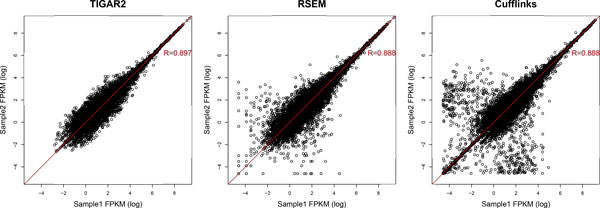
**Correlation of gene expression levels estimated from technical replicates**. Scatter plots of gene expression levels estimated from technical replicates produced from the Ion Torrent PGM RNA-Seq data. The Pearson correlation coefficients are calculated and shown on each plot. Predictions with TIGAR2 were most consistent among technical replicates.

**Figure 7:**
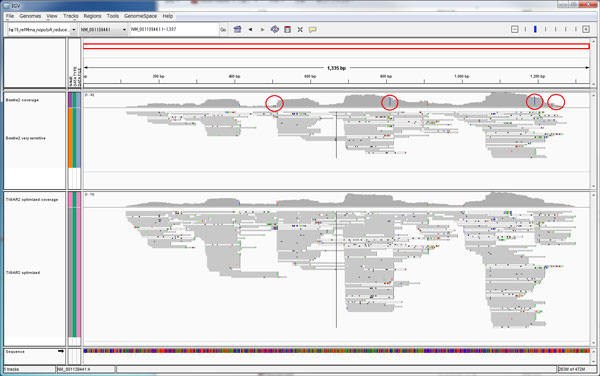
**Visualization of the optimized read alignment**. Read alignment on NM_001139441, which is an isoform of BAP31, is visualized by IGV. The top track shows the read alignment by Bowtie2, and the bottom track shows the optimized read alignment with TIGAR2. It became easier to identify possible sequencing errors from genetic variants by optimization with TIGAR2 (red circles).

To evaluate performance with TIGAR2 for real RNA-Seq data analysis, we obtained 4.25 million single-end reads of variable lengths of the human HeLa cell, which is publicly available from the Life Technologies' web site (http://ioncommunity.lifetechnologies.com). The sequencing was performed with the Ion PGM sequencer, which detects the protons released sequentially when one of the four nucleotide bases is introduced in real-time [18]. We divided the RNA-Seq data into two data sets, assuming that they are technical replicates obtained from the same experimental conditions. Gene expression levels were estimated with TIGAR2, RSEM, and Cufflinks and plotted in Figure 6 (the Pearson correlation coefficients of the estimated abundances between the two technical replicates were 0.897, 0.888 and 0.888, respectively). The result shows that the quantification with TIGAR2 was most consistent among the technical replicates, compared to those with RSEM and Cufflinks. TIGAR2 outputs the optimized read alignment on cDNA references in BAM format after inference is done, so that predicted isoforms can be followed up. The resultant BAM file can be loaded into a genome browser, such as Integrative Genomics Viewer [19]. This function is also a new feature that is not available in the original TIGAR and TopHat-Cufflinks. The bottom track in Figure 7 shows the optimized read alignments estimated with TIGAR2 for NM_001139441, which is an isoform of BAP31 that is known to be expressed in HeLa cells [20]. Compared to the read alignment by Bowtie2 (the top track in Figure 7), not only the amount of reads assigned to the isoform increased, but also it became easier to identify possible sequencing errors from genomic variants.

### Computational resources

CPU time and memory required in the real data analysis are summarized in Table 1. TIGAR2 was the fastest among others, notably more than two times faster than TIGAR1 with practical memory requirement. TopHat-Cufflinks was slower than TIGAR2, TIGAR1 and RSEM, especially in the alignment step.

**Table 1:** Computational resources required for the real data analysis

Tool	Implementation	CPU time in alignment (minutes)	CPU time in estimation (minutes)	Memory (GB)
TIGAR2	Java	18	12	8
TIGAR1	Java	18	53	8
RSEM	C++	10	24	2
TopHat-Cufflinks	C++	1,379	5	4

The CPU time and memory required for the real data analysis with TIGAR2, TIGAR1, RSEM, and TopHat-Cufflinks are summarized.

To see the scalability of TIGAR2 for a large dataset, it is applied to 100 million synthetic reads (100 bp single-end). It required 16 GB memory and 2,621 minutes of CPU time.

All the experiments were performed on an Intel Xeon CPU E5-2670 processor (2.60GHz) with the Red Hat Enterprise Linux Server release 6.2.

## Conclusions

We have developed a computational method, named TIGAR2, which is accurate and sensitive in quantifying gene expression levels of transcript isoforms from RNA-Seq data. TIGAR2 outperformed existing methods with simulation data of both single-end and paired-end reads (100 bp, 250 bp, 500 bp and 1000 bp), especially for reads > 250 bp. TIGAR2 will be more effective for accurate detection and quantification of transcript isoforms compared to other existing methods, as new technologies for longer sequencing become available.

Instead of trying to find novel transcript isoforms from RNA-Seq data, reference cDNA sequences of transcript isoforms are assumed to be known in the TIGAR2 pipeline. Although there are a couple of algorithms to predict novel transcript isoforms or fusion genes [2,14,21], TIGAR2 does not provide the novel predictions at the moment. However, once candidates of novel transcript isoforms are predicted by external tools, they can be treated as known and gene expression levels of these novel isoforms can be quantified and assessed with TIGAR2. Another possible extension of TIGAR2 includes modelling of underlying genomic variation for identifying allele-specific gene expression. Because the cost of whole genome-sequencing is dropping sharply, it is becoming feasible to use both genomic information as well as gene expression data. Finally, there should be an optimal balance between the maximum number of allowed alignments per read and the convergence speed. These topics will be investigated as our future works.

## Availability of supporting data

The implementation of TIGAR2 and the documentation is available in the GitHub repository, https://github.com/nariai/tigar2.

## Competing interests

The authors declare that they have no competing interests.

## Authors' contributions

NN and MN conceived the study, NN, KK, TM, and MN designed the computational experiments, NN performed the analysis, and NN, KK, TM, and MN interpreted the results. YS, YK, and YYK collaborated on data collection and interpretation of the results. NN, KK, TM, YS, YK, YYK and MN wrote the manuscript. All the authors read and approved the final manuscript.

## Declarations

The publication costs for this article were partly funded by MEXT Tohoku Medical Megabank Project and CREST.

This article has been published as part of *BMC Genomics *Volume 15 Supplement 10, 2014: Proceedings of the 25th International Conference on Genome Informatics (GIW/ISCB-Asia): Genomics. The full contents of the supplement are available online at http://www.biomedcentral.com/bmcgenomics/supplements/15/S10.
